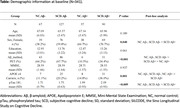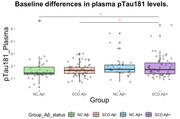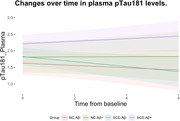# Plasma ptau181 levels at baseline and over time according to amyloid status and SCD in SILCODE cognitively unimpaired older adults

**DOI:** 10.1002/alz.091100

**Published:** 2025-01-09

**Authors:** Kai Shao, Elizabeth Kuhn, Xiaochen Hu, Shaozhen Yan, Zhigeng Chen, Beiqi He, Xueyan Jiang, Ruixian Li, Xuanqian Wang, Min Wei, Yongzhe Wei, Jie Yang, Xianfeng Yu, Mingkai Zhang, Liang Zhang, Jie Lu, Michael Wagner, Ying Han, SILCODE study group

**Affiliations:** ^1^ XuanWu Hospital of Capital Medical University, Beijing China; ^2^ German Center for Neurodegenerative Diseases (DZNE), Bonn Germany; ^3^ Department of Psychiatry, University of Cologne, Medical Faculty, Cologne Germany; ^4^ University of Bonn Medical Center, Dept. of Neurodegenerative Disease and Geriatric Psychiatry/Psychiatry, Bonn Germany; ^5^ German Center for Neurodegenerative Diseases (DZNE), Bonn/Cologne Germany; ^6^ Department of Radiology and Nuclear Medicine, XuanWu Hospital of Capital Medical University, Beijing China; ^7^ School of Information and Communication Engineering, Hainan University, Haikou China; ^8^ State key laboratory of digital medical engineering, School of Biomedical Engineering, Hainan University, Sanya China; ^9^ National Clinical Research Center for Geriatric Disorders, Beijing China; ^10^ Center of Alzheimer’s Disease, Beijing Institute for Brain Disorders, Beijing China; ^11^ School of Biomedical Engineering, Hainan University, Haikou China; ^12^ Institute of Biomedical Engineering, Shenzhen Bay Laboratory, Shenzhen China

## Abstract

**Background:**

Cognitively unimpaired (CU) older adults with abnormal levels of β‐amyloid (Aβ) deposition are considered in the preclinical stage of Alzheimer’s disease (AD) and, when combined with the subjective cognitive decline (SCD), are proposed as the stage 2 AD in the NIA‐AA framework. Here, we aim to investigate whether neuropathologic deterioration increases in early stages, particularly in stage 2 AD.

**Method:**

We included 341 CU participants over 50 years of age from the Sino Longitudinal Study on Cognitive Decline (SILCODE) study. SCD was defined based on baseline cognitive complaints with associated concern/worry, whereas normal control (NC) did not express any concern/worry. Aβ status was defined based on amyloid‐PET or, if not available, on plasma Aβ42/40 ratio. All selected participants had available plasma phospho‐tau181 (ptau181), and were divided into four groups according to SCD combined with baseline Aβ positivity. General linear models and linear mixed‐effect models were performed to determine whether ptau181 levels differed between groups (i.e., NC.Aβ‐, SCD.Aβ‐, NC.Aβ+, SCD.Aβ+) at baseline and over time.

**Result:**

At baseline, the four groups were significantly different in terms of ptau181 levels (p=0.001), with the SCD.Aβ+ group having higher levels than NC.Aβ‐ (p=0.004) and SCD.Aβ‐ (p=0.003), but not different from NC.Aβ+ (p=0.63). In the smallest sample of 81 CU with available follow‐up for ptau181 (i.e., 15 NC.Aβ‐, 35 SCD.Aβ‐, 8 NC.Aβ+, 23 SCD.Aβ+), we replicated the baseline differences (p=0.037). Longitudinal analysis showed that ptau181 levels changed differently between groups over time (p=0.016), with SCD.Aβ+ showing a faster increase over time than SCD.Aβ‐ (p=0.011).

**Conclusion:**

Our results showed that participants with stage 2 AD (SCD.Aβ+) have a higher ptau181 burden at baseline and increasing plasma ptau181 levels over time in a Chinese sample. This suggests that this group is at higher risk for neuropathologic changes, although no differences were found with stage 1 (NC.Aβ+). It should be noted that sample sizes for longitudinal analyses were small, especially for NC, which may partly explain this lack of differences between stages 1 and 2 AD. The SILCODE follow‐up is ongoing, and we plan to test this hypothesis at a later stage.